# Scrofula

**Published:** 1860-10

**Authors:** 


*Scrofula.*—Dr. Gregory, of Edinburgh, asserts as his belief that there is not a family in Great Britain in which scrofula does not exist. B.

